# An observational study of centrally facilitated pain in individuals with chronic low back pain

**DOI:** 10.1097/PR9.0000000000001003

**Published:** 2022-04-13

**Authors:** Vasileios Georgopoulos, Kehinde Akin-Akinyosoye, Stephanie Smith, Daniel F. McWilliams, Paul Hendrick, David A. Walsh

**Affiliations:** a Academic Rheumatology, School of Medicine, University of Nottingham, Nottingham, United Kingdom; b Pain Centre Versus Arthritis, University of Nottingham, Nottingham, United Kingdom; c NIHR Nottingham BRC, University of Nottingham, Nottingham, United Kingdom; d Physiotherapy, School of Health Sciences, University of Nottingham, Nottingham, United Kingdom

**Keywords:** Central pain hypersensitivity, Pain, Low back pain, Prospective study, Outcome prediction

## Abstract

**Introduction:**

Central pain facilitation can hinder recovery in people with chronic low back pain (CLBP).

**Objectives:**

The objective of this observational study was to investigate whether indices of centrally facilitated pain are associated with pain outcomes in a hospital-based cohort of individuals with CLBP undertaking a pain management programme.

**Methods:**

Participants provided self-report and pain sensitivity data at baseline (n = 97) and again 3 months (n = 87) after a cognitive behavioural therapy–based group intervention including physiotherapy. Indices of centrally facilitated pain were pressure pain detection threshold, temporal summation and conditioned pain modulation at the forearm, Widespread Pain Index (WPI) classified using a body manikin, and a Central Mechanisms Trait (CMT) factor derived from 8 self-reported characteristics of anxiety, depression, neuropathic pain, fatigue, cognitive dysfunction, pain distribution, catastrophizing, and sleep. Pain severity was a composite factor derived from Numerical Rating Scales. Cross-sectional and longitudinal regression models were adjusted for age and sex.

**Results:**

Baseline CMT and WPI each was associated with higher pain severity (CMT: *r* = 0.50, *P* < 0.001; WPI: *r* = 0.21, *P* = 0.04) at baseline and at 3 months (CMT: *r* = 0.38, *P* < 0.001; WPI: *r* = 0.24, *P* = 0.02). High baseline CMT remained significantly associated with pain at 3 months after additional adjustment for baseline pain (β = 2.45, *P* = 0.04, *R*^2^ = 0.25, *P* < 0.0001). Quantitative sensory testing indices of pain hypersensitivity were not significantly associated with pain outcomes at baseline or at 3 months.

**Conclusion:**

Central mechanisms beyond those captured by quantitative sensory testing are associated with poor CLBP outcome and might be targets for improved therapy.

## Abstract 2

Supplemental Digital Content is Available in the Text.

Indices of centrally facilitated pain, collected early in rehabilitation pathways, are associated with worse pain severity at follow-up. Appropriate early reduction of pain hypersensitivity might improve such outcomes.

## 1. Introduction

Chronic low back pain (CLBP) is the most prevalent musculoskeletal condition that significantly affects on quality of life and health care services.^70^ In chronic pain, nociceptive signals from peripheral tissues occur in parallel with maladaptive processing within the central nervous system.^5^ Peripheral and central nervous system processing contributes to the severity and persistence of CLBP,^7,12^ constituting “central pain facilitation.” The central mechanisms that amplify CLBP are not fully understood. With central sensitisation, central neurones have increased responsiveness to peripheral nociceptive drive.^33^ Central pain facilitation may result from increased connectivity between sensory and emotional control regions in the brain and decreased connectivity with descending inhibitory pathways.^3^ Cognition, emotion, motivation, and localisation^54^ contribute to the multidimensional experience of pain and drive-related behavioural responses,^45^ and alterations in central neuronal processing might underlie problems with anxiety or depression (negative affect),^1,11^ cognition,^1^ and fatigue,^1,66^ each of which contributes adversely to the pain experience.

Evidence of centrally facilitated pain is consistently found in CLBP.^7,55^ Central facilitation increases severity and impact of chronic pain and may pose barriers to recovery when using peripherally targeted treatments.^12,18,24,35,49,53^ Evidence-based guidelines recommend cognitive behavioural therapy (CBT)–based group interventions including physiotherapy for people with CLBP,^50^ which may address aspects of central pain facilitation. It is unknown whether central pain facilitation predicts or poses a barrier to pain improvement within this therapeutic context.

Quantitative sensory testing (QST) can indicate central pain hypersensitivity and provide insights into pain mechanisms. Static (eg, pain pressure detection thresholds [PPT] and applied distant to the site of pathology) or dynamic (temporal summation [TS] and conditioned pain modulation [CPM]) QST modalities assess different aspects of central pain processing.^7,61,80^ Indices of centrally facilitated pain have been associated with negative effect (anxiety and depression), catastrophizing, neuropathic-like pain, fatigue, sleep disturbance, pain distribution, and cognitive impact in people with musculoskeletal pain.^1,11,14,31,66^ Such characteristics have been implicated also in increased pain severity in individuals with CLBP.^32,43,61,62^ Items that display face validity as measures of central mechanisms, selected from questionnaires that address these 8 characteristics, can together measure a latent Central Mechanisms Trait (CMT) factor associated with QST evidence of central pain hypersensitivity in individuals with knee pain.^1^ The contribution of these characteristics to a latent trait in a population with low back pain has not been previously established. Pain distribution, self-reported on a body manikin, beyond sites of tissue injury, may itself identify people with centrally facilitated pain.^16,74,77^

We hypothesised that self-reported and QST indices of centrally facilitated pain are associated with higher pain severity in people with CLBP after a CBT-based pain management programme involving physiotherapy.

## 2. Methods

### 2.1. Participants and study design

We report here an analysis of pain outcome data from an observational, prospective cohort study, whose primary objective was to ascertain whether indices of centrally facilitated pain predict self-management outcomes (ClinicalTrials.gov: NCT03972332). Individuals with CLBP were enrolled on day 1 (baseline) of their participation in a group intervention programme, which aimed to facilitate self-management and self-care. Participants undertook clinical examination including QST and completed a questionnaire booklet which included self-reported tools about pain severity and comorbidities at baseline (before or on the first day of their intervention) and approximately 3 months after baseline. Participants were recruited within Nottinghamshire, United Kingdom, between May 2018 and August 2019, through the Back Pain Unit of Sherwood Forest Hospitals NHS Foundation Trust, pain services of the Primary Integrated Community Services, and the Nottingham CityCare Partnership. Approvals were obtained from the East Midlands–Nottingham 1 Research Ethics Committee of the Health Research Authority, United Kingdom (REC: 18/EM/0049).

### 2.2. Therapeutic context

A therapeutic context targeting biopsychosocial aspects of pain was selected. All recruited individuals were newly enrolled participants in a CBT-based group intervention programme, delivered either by a physiotherapist (PT) or multidisciplinary team (MDT). Programme allocation was by a clinical team independent of this study, in liaison with the patient. Patients with relatively recent onset of CLBP (3–12 months), moderate to low levels of average daily pain (numerical rating scales [NRS] ≤ 4), and disability or emotional distress were eligible for the PT group intervention programme. Details about programme allocation and programme content are given in the Supplementary Methods (available at http://links.lww.com/PR9/A158).

### 2.3. Inclusion and exclusion criteria

All programme participants during the study period were screened for inclusion in this study. Individuals were eligible for programme participation if they were adults (older than 18 years), had the ability to give informed consent, were diagnosed with CLBP and reported the lumbar region as the index site of pain, were enlisted for participation in a pain management programme, and were able to speak and understand English. Individuals were excluded if they were pregnant; unable to give informed consent or understand key aspects of the study because of cognitive impairment; or gave a history of additional comorbidities such as cancer, diabetic neuropathies, fractures, or other conditions causing greater disability than their back pain.

### 2.4. Assessment of pain severity

Back pain severity was assessed with the pain or discomfort dimension of the EQ-5D-5L^30^ and four 11-point NRS.^22,72^ EQ-5D-5L measures the level of pain or discomfort today (0-no problem, 5-extremely severe). The 4 NRS rated pain today, current pain, strongest pain over the last 4 weeks, and average pain over the last 4 weeks, with 0 indicating no pain and 10 the worst pain imaginable. A single pain severity index (pain factor) was derived from these 5 items by using confirmatory factor analysis.

### 2.5. Indices of centrally facilitated pain

#### 2.5.1. Quantitative sensory testing

Quantitative sensory testing comprised both “static” (PPT) and “dynamic” (TS and CPM) modalities.^7,60,79^ Test sites were localised from anatomical landmarks and marked with a pen to ensure consistency between repeated stimulations. The brachioradialis muscle, approximately 5 cm distal to the lateral epicondyle,^60^ was chosen for all modalities as a site distant from the primary area of pain in individuals with CLBP. All QST was undertaken by a single observer (V.G.), and participants were requested to have their eyes closed. Intraclass correlation coefficients (ICC) for 25 participants showed moderate-to-good repeatability with a mean test–retest interval of 8 (SD ± 1) days (PPT: 0.92; 95% CI: 0.83–0.96, TS: 0.78; 95% CI: 0.56–0.86, CPM: 0.44; 95% CI: 0.07–0.71). Participants were excluded from QST assessment if they reported, or, on clinical examination, displayed pain originating from the neck, shoulder, elbow, or forearm.

##### 2.5.1.1. Forearm pressure pain detection threshold

Pain pressure detection thresholds were measured using a handheld digital algometer (Medoc-AlgoMed Advanced Medical Systems—Computerised Pressure Algometer, Israel). A 1-cm diameter probe was held perpendicular to the skin and force applied at a constant incremental rate of 50 kPa/second. Participants were instructed to activate a handheld device when the sensation of pressure became painful. PPT was taken as the arithmetic mean of 3 replicate measurements at the test site. Low PPT indicated greater pain sensitivity.

##### 2.5.1.2. Temporal summation

Pain TS was assessed twice by repeated application to the forearm of a punctate stimulus (256 mN) using the retractable blunt needle of a specially manufactured pen (MRC Systems GmbH; The Pin Prick, Germany), while the participant sat comfortably on an examination plinth (Addax Practice Manager—3 Section Electric Treatment Couch, United Kingdom). A single punctate stimulus was applied on their dominant forearm, followed by 10 repetitive stimuli at a rate of 1/s.^6^ Immediately after the single stimulus, and after the 10 repeated stimuli, each participant was asked to rate the experienced intensity of pain or sharpness (single sensation for single stimulus and average of 10 for repeated stimuli, respectively) on a paper copy of a 10-cm visual analogue scale. Temporal summation was calculated as windup difference (TS^WUD^ = average of 10 stimuli − single stimulus). The average of the 2 TS^WUD^ values was used for analysis. Larger positive values of TS indicated greater sensitivity.

##### 2.5.1.3. Conditioned pain modulation

For the purposes of CPM, the participant's unconditioned PPT was the arithmetic mean of the 3 replicate measurements, assessed earlier on their nondominant forearm (see above). The conditioned PPT was assessed using contralateral forearm ischaemic pain as the conditioning stimulus, rated as 4 on an 11-point current pain NRS. The participant's dominant arm was compressed to occlude arterial blood flow (absent brachial pulse) by progressive inflation of a 15-cm cuff similar to those used to measure blood pressure. Intensity of the conditioning pain or discomfort was limited to no greater than 4 on an 11-point NRS.^79^ When pain was reported as <4/10 in the absence of brachial pulse, participants were then asked to squeeze a foam ball of a tennis ball size continuously until they rated their pain or discomfort in the dominant (ball-holding) arm at 4/10. The conditioned PPT was then assessed by a single application of the algometer over brachioradialis in their nondominant forearm, after which the pressure cuff was immediately released. CPM was taken to be the single conditioned PPT measurement (PPT^Con^) minus the arithmetic mean of the replicate unconditioned PPT measurements (PPT^Mean^) (CPM = PPT^Con^—PPT^Mean^).^78,79^ A lower positive or more negative CPM value indicated higher sensitivity (less efficient CPM).^44^

#### 2.5.2. Pain distribution

Pain distribution was self-reported using a body manikin^1^ coded in 24 sites (Supplementary Fig. 1, available at http://links.lww.com/PR9/A158)^16^ and classified according to the Widespread Pain Index (WPI) criteria.^74^

#### 2.5.3. Central Mechanisms Trait

Eight items measuring anxiety, catastrophizing, cognitive impairment, depression, fatigue, neuropathic-like pain, pain distribution, and sleep (Table 1) have each been found to contribute to a single CMT factor in people with knee pain.^1^ To replicate the trait validated previously for knee pain and to confirm its validity in a population with low back pain, a modified single Central Mechanisms Trait factor was calculated from 8 items taken from self-reported measures of people who participated in this study. Items for low back pain, where not identical to those for knee pain, were chosen based on textual or contextual similarity. Receiver operating characteristics analysis established the threshold number of reported pain sites which optimally classified forearm PPT in the lowest quartile of the study population.^1^ Pain distribution was then classified as above or below that threshold for calculation of the Central Mechanisms Trait.

**Table 1:** Items comprising the Central Mechanisms Trait.

Characteristic	Validated items used for knee pain (Ref. 1)		Adapted items used for low back pain	
	Originating questionnaire	Item text	Originating questionnaire	Item text
1. Neuropathic-like pain	painDETECT questionnaire [2]	Is cold or heat (bath water) in this area occasionally painful? (possible range 0–5)	painDETECT questionnaire [2]	Is cold or heat (bath water) in this area occasionally painful? (possible range 0–5)
2. Anxiety	Hospital Anxiety and Depression Scale—Anxiety Subscale [10]	I get sudden feelings of panic (possible range 0–3)	Hospital Anxiety and Depression Scale—Anxiety Subscale [10]	I get sudden feelings of panic (possible range 0–3)
3. Depression	Hospital Anxiety and Depression Scale—Depression Subscale [10]	I still enjoy the things I used to enjoy (possible range 0–3)	Hospital Anxiety and Depression Scale—Depression Subscale [10]	I still enjoy the things I used to enjoy (possible range 0–3)
4. Cognitive impact	**Measured by a single item [7]**	**Does your pain or other bodily symptoms stop you from concentrating on what you are doing? (possible range 0–4)**	**Fibromyalgia Severity Scale [9]**	**Please could you indicate your level of concentration problems (forgetfulness and problem solving) severity score over the past week? (possible range 0–3)**
5. Catastrophizing	Pain Catastrophizing Scale [8]	I keep thinking about how much it hurts (possible range 0–4)	Pain Catastrophizing Scale [8]	I keep thinking about how much it hurts (possible range 0–4)
6. Sleep	**Intermittent and Constant OA Knee Pain—Constant Subscale [3]**	**In the past week, how much has your constant knee pain affected your sleep? (possible range 0–4)**	**Roland–Morris Disability Questionnaire [6]**	**I sleep less well because of my back (yes/no)**
7. Pain distribution	Body manikin [5]	This question is about recent pain you may have had in any part of your body. Please shade in the diagram below to indicate where you have suffered any pain for most days in the previous month. By pain, we also mean aching, discomfort, and/or stiffness. Please do not include pain due to feverish illness such as flu.	Body manikin [5]	This question is about recent pain you may have had in any part of your body. Please shade in the diagram below to indicate where you have suffered any pain for most days in the previous month. By pain, we also mean aching, discomfort, and/or stiffness. Please do not include pain due to feverish illness such as flu.
8. Fatigue	**Measured by a single item [7]**	**In the past month, did you feel tired on most days? (possible range 0–5)**	**Fatigue Severity Scale [4]**	**Total score (possible range 0–63)**

Items for low back pain, where not identical, were used as surrogates and were chosen based on textual or contextual similarity to those described by Akin-Akinyosoye et al.^1^ The sleep single item was replaced by a single item from the Roland–Morris Disability Questionnaire,^6^ the fatigue single item was replaced by the total score of the Fatigue Severity Scale,^4^ and the cognitive impact single item was replaced by a single item from the Fibromyalgia Severity Scale.^9^ Items and text differing between the previous and current work are highlighted with bold.

Table 1 references: [1] Akin-Akinyosoye K, Frowd N, Marshall L, Stocks J, Fernandes GS, Valdes A, McWilliams DF, Zhang W, Doherty M, Ferguson E, Walsh DA. Traits associated with central pain augmentation in the Knee Pain In the Community (KPIC) cohort. Pain 2018;159(6):1035. [2] Freynhagen R, Baron R, Gockel U, Tölle TR. Pain DETECT: a new screening questionnaire to identify neuropathic components in patients with back pain. Current medical research and opinion 2006;22(10):1911-1920. [3] Hawker G, Davis A, French M, Cibere J, Jordan J, March L, Suarez-Almazor M, Katz J, Dieppe P. Development and preliminary psychometric testing of a new OA pain measure–an OARSI/OMERACT initiative. Osteoarthritis and Cartilage 2008;16(4):409-414. [4] Krupp LB, LaRocca NG, Muir-Nash J, Steinberg AD. The fatigue severity scale: application to patients with multiple sclerosis and systemic lupus erythematosus. Archives of neurology 1989;46(10):1121-1123. [5] Lacey RJ, Lewis M, Jordan K, Jinks C, Sim J. Interrater reliability of scoring of pain drawings in a self-report health survey. Spine 2005;30(16):E455-E458. [6] Roland M, Morris R. A study of the natural history of back pain: part I: development of a reliable and sensitive measure of disability in low-back pain. spine 1983;8(2):141-144. [7] Sirri L, Grandi S, Fava GA. The illness attitude scales. Psychotherapy and Psychosomatics 2008;77(6):337-350. [8] Sullivan MJ, Bishop SR, Pivik J. The pain catastrophizing scale: development and validation. Psychological assessment 1995;7(4):524. [9] Wolfe F, Clauw DJ, Fitzcharles M-A, Goldenberg DL, Häuser W, Katz RL, Mease PJ, Russell AS, Russell IJ, Walitt B. 2016 Revisions to the 2010/2011 fibromyalgia diagnostic criteria, Proceedings of the Seminars in arthritis and rheumatism, Vol. 46: Elsevier, 2016. pp. 319-329. [10] Zigmond AS, Snaith RP. The hospital anxiety and depression scale. Acta psychiatrica scandinavica 1983;67(6):361-370.

Additional details about indices of centrally facilitated pain are in Supplementary Methods (available at http://links.lww.com/PR9/A158).

### 2.6. Clinical characteristics

Neuropathic characteristics of CLBP were assessed with the painDETECT questionnaire.^22^ Anxiety and depression were assessed with the Hospital Anxiety and Depression Scale,^82^ catastrophization was assessed with the Pain Catastrophization Scale,^67^ and fatigue with the Fatigue Severity Scale.^40^ Disability was assessed with the Roland–Morris Disability Questionnaire,^59^ and severity of fibromyalgia-like symptoms was assessed with the Fibromyalgia Severity Scale.^74^

Additional details about the measurement of clinical characteristics are in Supplementary Methods (available at http://links.lww.com/PR9/A158).

### 2.7. Analysis

Presented data are means ± SD or median with interquartile range (IQR). Unadjusted associations are presented as Pearson product-moment (*r*) or Spearman rank-order (ρ) correlation coefficients. Associations were considered little or zero, fair, moderate to good, and good to excellent when r values were between 0.00 to 0.25, 0.25 to 0.50, 0.50 to 0.75, and >0.75, respectively.^56^

Confirmatory factor analysis was used to fit data to a single pain factor score from the 5 pain severity outcome measures (Supplementary Fig. 2, available at http://links.lww.com/PR9/A158)^58,71^ and a single Central Mechanisms Trait score from 8 self-reported items (Supplementary Fig. 3, available at http://links.lww.com/PR9/A158).^1^ Confirmation of model fit was based on the root mean square error of approximation (RMSEA) fit index.^19^ Root mean square error of approximation values of <0.05 constitute good fit, 0.05 to 0.08 acceptable fit, 0.08 to 0.10 marginal fit, and >0.10 poor fit.^13^ Additional values indicative of model fit were the Comparative Fit Index (CFI) ≥0.95, Tucker–Lewis Index (TLI) ≥0.95, χ^2^ *P*-value ≤0.05, and standardised root mean square residual (SRMR) ≥0.07.^81^

In regression modelling, pain factor was the dependent variable (either at baseline or at follow-up), and the independent variables comprised indices of centrally facilitated pain (QST modalities, pain distribution, and the Central Mechanisms Trait) and demographic variables (age and sex) previously found to predict increased pain severity.^2,46^ Separate models were explored for each index of centrally facilitated pain, each adjusted for age and sex. As depression, catastrophizing and fatigue are characteristics which contributed to the Central Mechanisms Trait score; these variables were not included in the model. Baseline pain factor was included as an additional independent variable when examining pain at follow-up, to explore possible barriers to improvement in pain (follow-up pain adjusted for baseline pain indicates the magnitude of change in pain). Goodness of model fit and the explanatory power of regression models were evaluated using coefficient of determination (adjusted *R*^2^).^36^ Multicollinearity was evaluated using variance inflation factor.^25,36^ Correlation coefficients and regression coefficients were adjusted after multiple comparisons according to Benjamini and Hochberg.^34^

All analyses used R (version 3.4.2),^57^ and *P*-values of ≤0.05, after adjusted for multiple comparisons, were taken to indicate statistical significance. Significant correlations or associations are indicated by bold font in tables. Post hoc power calculations were conducted with G*Power software (version 3.1.9.7).^20^ Details about data handling and effect size calculation are reported in Supplementary Methods (available at http://links.lww.com/PR9/A158).

## 3. Results

### 3.1. Demographic and clinical characteristics

**Figure 1.**
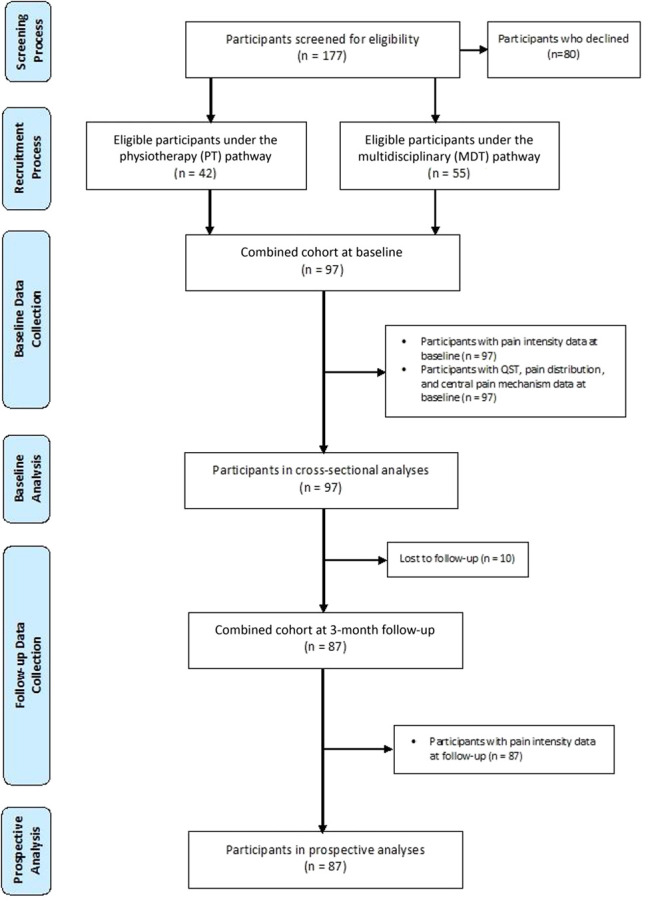
Flow diagram of the eligibility screening, recruitment, and data collection processes.

Participant recruitment and retention are given in Figure 1. Of 177 eligible individuals with CLBP, 97 (71% female participants, mean age 56 ± 13 years) contributed baseline data, whereas 80 (70% female participants, mean age 54 ± 14 years) declined participation. No eligible participants were excluded because of upper limb or cervical pathology. Nine participants (9.3%) reported forearm pain on the manikin. Study participants engaged in a median of 9 (IQR: 8–10) of the 10 MDT sessions or 5 (IQR: 4–5) of the 5 PT intervention sessions.

Table 2 gives baseline demographic and clinical characteristics, and Table 3 gives baseline and follow-up pain severity. The 87 participants (PT: n = 39, 51% female participants, mean age 58 ± 13 years; MDT: n = 48, 79% female participants, mean age 56 ± 13 years) who provided follow-up data (mean age 57 (±13) years, BMI 29.4 (26.0–34.5) kg/m^2^, 67% female) and 10 participants lost to follow-up (mean age 49 (±16) years, BMI 29.1 (22.4–39.5) kg/m^2^, 50% female), each displayed similar characteristics to the total study population. Mean or median baseline scores indicated moderate pain severity, depressive symptoms, anxiety, and catastrophizing.

**Table 2:** Participant demographics and clinical characteristics at baseline.

Characteristic (possible range)	Baseline
Number of participants	97
Physiotherapy lead programme	42
Multidisciplinary lead programme	55
Age (y)	56 (±13)
Physiotherapy lead programme	57 (±13)
Multidisciplinary lead programme	55 (±14)
BMI (kg/m^2^)	29.4 (25.7–34.6)
Female	63 (71%)
Physiotherapy lead programme	22 (52%)
Multidisciplinary lead programme	41 (75%)
Setting	
Hospital	92 (95%)
Community	5 (5%)
Self-reported clinical characteristics	
painDETECT (0–38)	17 (12–24)
Hospital Anxiety Scale (0–21)	9 (6–13)
Hospital Depression Scale (0–21)	9 (5–12)
Pain Catastrophizing Scale (0–52)	22 (11–31)
Roland–Morris Disability Questionnaire (0–24)	13 (9–18)
Fatigue Severity Scale (7–63)	42 (29–52)
Fibromyalgia Severity Scale (0–31)	13 (8–18)
Quantitative sensory testing	
Pain pressure detection threshold (kPa)	205.8 (148.2–297.6)
Temporal summation (0–10)	1.0 (0.4–2.8)
Conditioned pain modulation (kPa)	59.1 (5.6–99.3)
Widespread Pain Index (present)*	35 (36%)
Central Mechanisms Trait factor (−1.2 to 1.4)	0.05 (−0.45–0.43)
Types of medication†	
Nonsteroidal anti-inflammatory drugs	74 (76%)
Opioids	60 (62%)
Neuromodulators	64 (66%)
Topical analgesics	4 (4%)

Data are presented as mean (± SD), median (Interquartile range), or n (%).

* Reflects the number and percentage of participants satisfying criteria to be classified as demonstrating widespread pain.

† Reflects the number and percentage of participants used each type pf medication. One participant could use more than 1 type of medication.

BMI, body mass index; kPa, kilopascals.

**Table 3:** Patient-reported pain outcomes at baseline and 3-mo follow-up.

Characteristic (possible range)	Baseline (n = 97)	3 Months (n = 87)	Change	Change significance* WSRT (*P*)	Effect size†
Pain constructs					
NRS (0–10)	6 (5 to 7)	5 (4 to 7)	−1 (−2 to 0)	**1765 (<0.01)**	−0.4
PD _Now_ (0–10)	6 (4 to 7)	5 (3 to 7)	−1 (−2 to 1)	**1794 (<0.01)**	−0.4
PD _Strongest_ (0–10)	8 (8 to 9)	8 (7 to 9)	0 (−1 to 0)	**991 (0.01)**	−0.3
PD _Average_ (0–10)	6 (6 to 7)	6 (5 to 7)	−1 (−2 to 0)	**1471 (0.01)**	−0.3
EQ4 _Pain/Discomfort_ (1–5)	3 (3 to 4)	3 (3 to 4)	0 (0 to 1)	**363 (<0.01)**	−0.3
Pain factor (−22 to 12)	0.31 (−2.68 to 3.61)	−1.38 (−6.23 to 1.82)	−2.20 (−5.05 to 0.95)	**2806 (<0.001)**	−0.43

Data are presented as median (interquartile range). Values in bold indicate statistical significance (*P* < 0.05).

* **C**hange significance; probability that the observed change between baseline and follow-up might have occurred by chance, assessed by the paired Wilcoxon signed-rank test (*P*-value).

† Effect size calculated as difference between baseline and follow-up divided by the SD at baseline.

EQ4 _Pain/Discomfort_, EQ-5D-5L Pain/Discomfort Today Domain; MCID, minimum clinically important difference; NRS, Numerical Rating Scale; PD _Average_, painDETECT Average Pain Scale (past 4 weeks); PD _Now_, painDETECT Pain Now Scale; PD _Strongest_, painDETECT Strongest Pain Scale (past 4 weeks); WSRT, Wilcoxon signed-rank test (paired).

Nine or more painful sites on the 24-site manikin optimally classified lower quartile baseline PPT at brachioradialis (AUC; 0.67, 95% CI: 0.55–0.80, *P* < 0.001) (Supplementary Fig. 4, available at http://links.lww.com/PR9/A158). Sensitivity analysis excluding those reporting pretest forearm pain at the PPT test site similarly indicated a 9/24 threshold. This threshold was therefore used as the pain distribution item for calculating CMT scores. Confirmatory factor analyses indicated that each of the 5 pain severity measures significantly loaded on a single “pain factor” (loading values; 0.62–0.91), and each of the 8 self-reported central mechanisms items significantly loaded on a single “Central Mechanisms Trait” factor (loading values; 0.33–0.76). Data fit to single pain factor and Central Mechanisms Trait models: pain factor—CFI = 0.98, TLI = 0.81; RMSEA = 0.24; SRMR = 0.07; χ^2^(*df*) = 33.84(20), *P* < 0.001; Central Mechanisms Trait factor—CFI = 0.92, TLI = 0.88; RMSEA = 0.08; SRMR = 0.07; χ^2^(*df*) = 34.19(20), *P* = 0.03).

Overall, at 3-month follow-up, pain factor and each of its component items demonstrated small but significant improvements from baseline (median single item improvements −1 to 0, median pain factor improvement −2.20 (scale range −22 to 12) (Table 3). No significant multicollinearity was detected between any combination of independent variables in cross-sectional or longitudinal analyses (variance inflation factor = 1.19–2.50). Residuals were normally distributed in all examined models (Shapiro–Wilk *P* > 0.05).

### 3.2. Cross-sectional associations between baseline indices of centrally facilitated pain and pain severity

Indices of centrally facilitated pain were intercorrelated in the expected direction (Table 4). Low PPT was associated with greater TS (*r* = −0.40, *P* < 0.01) and higher CMT (*r* = −0.19, *P* = 0.03), and lower CPM was associated with greater TS (*r* = −0.22, *P* = 0.03). More widespread pain and higher Central Mechanisms Trait were associated with higher pain factor (WPI: *r* = 0.21, *P* = 0.04; CMT: *r* = 0.50, *P* < 0.001) (Table 4). Women and younger participants at baseline displayed higher indices of central pain hypersensitivity (Supplementary Table 1, available at http://links.lww.com/PR9/A158).

**Table 4:** Correlation matrix between pain and indices of centrally facilitated pain at baseline.

Pain index	PPT (kPa)		TS (0–10)		CPM (kPa)		WPI (yes/no)		CMT (index)	
	Cor	*P*	Cor	*P*	Cor	*P*	Cor	*P*	Cor	*P*
TS (0–10)	**−0.40**	**<0.01**								
CPM (kPa)	0.12	0.22	**−0.22**	**0.03**						
WPI (yes/no)	−0.14	0.24	−0.06	0.64	−0.12	0.35				
CMT (index)	**−0.19**	**0.03**	0.13	0.22	−0.02	0.84	**0.37**	**<0.01**		
Pain factor	−0.06	0.59	0.11	0.30	0.03	0.76	**0.21**	**0.04**	**0.50**	**<0.001**

Data are from n = 97 participants. All *P*-values have been corrected for multiple comparisons (Benjamini–Hochberg). Values in bold indicate statistical significance (*P* < 0.05).

CMT, Central Mechanisms Trait; Cor, Spearman rank-order correlation; CPM, conditioned pain modulation; PPT, pain pressure detection threshold; TS, temporal summation; WPI, Widespread Pain Index.

Baseline Central Mechanisms Trait (Tables 4 and 5) and each of the 8 contributing characteristics were significantly associated with pain factor (Supplementary Table 2, available at http://links.lww.com/PR9/A158), in both bivariate correlations and multivariable regression models adjusted for age and sex. Higher baseline WPI, but not baseline QST modalities, was significantly associated with pain factor at baseline.

**Table 5:** Multivariable models exploring the cross-sectional relationship between baseline measurements of indices of centrally facilitated pain and pain severity.

Baseline variables (primary predictor)	Baseline pain factor				
	Bivariate		Adjusted for age and sex		
	B	*P*	β	SE	*P*
QST					
PPT (kPa)	−0.21	0.63	−0.36	0.46	0.44
Adjusted *R*^2^*(P)*	—		−0.01 (0.64)		
TS (0–10)	5.84	0.30	6.98	5.68	0.22
Adjusted *R*^2^*(P)*	—		−0.01 (0.48)		
CPM (kPa)	0.002	0.77	0.001	0.01	0.89
Adjusted *R*^2^*(P)*	—		−0.02 (0.80)		
Widespread pain					
WPI (yes/no)	**2.09**	**0.04**	**2.17**	**1.05**	**0.04**
Adjusted *R*^2^*(P)*	—		0.02 (0.16)		
CMT					
CMT factor	**4.34**	**<0.0001**	**4.99**	**0.82**	**<0.0001**
Adjusted *R*^2^*(P)*	—		**0.26** (**<0.0001)**		

B values represent bivariate regressions between baseline variables and baseline pain factors, whereas β-values represent standardised regression coefficients for each listed baseline variable within multivariable regression models created for each central pain hypersensitivity index. Each multivariable model was adjusted for age and sex. Multicollinearity testing yielded VIF values ranging from 1.03 to 1.20 for all independent variables indicating not significant multicollinearity between them. Values calculated from baseline data of n = 97 participants. All *P*-values have been corrected for multiple comparisons (Benjamini–Hochberg). Values in bold indicate statistical significance.

CMT, Central Mechanisms Trait; CPM, conditioned pain modulation; PPT, pain pressure detection threshold; QST, quantitative sensory testing; TS, temporal summation; WPI, Widespread Pain Index.

### 3.3. Longitudinal associations between baseline indices of centrally facilitated pain and pain severity at follow-up

Details of bivariate and multivariable regression models showing longitudinal associations between the different baseline indices of centrally facilitated pain and pain factor at 3-month follow-up are provided in Table 6. The sample size for longitudinal analysis (n = 87) was sufficient for 99% power to explain 25% of the variance (*R*^2^ ≥ 0.25) in multivariable models featuring 4 variables (index of centrally facilitated pain, pain factor at baseline, age, and sex). In bivariate regressions, baseline CMT and WPI, but not QST modalities, were significantly associated with pain factor at 3-month follow-up (Supplementary Table 3, available at http://links.lww.com/PR9/A158). Pain factor at baseline was significantly correlated with its follow-up counterpart (*r* = 0.48, *P* < 0.0001). Association between higher baseline CMT and higher follow-up pain factor remained significant after adjustment for baseline pain factor, age, and sex (Table 6). These findings are expressed as associations between baseline CMT and change in pain factor from baseline to 3-month follow-up in Supplementary Table 3 (available at http://links.lww.com/PR9/A158). The other indices of centrally facilitated pain at baseline were not significantly associated with follow-up pain factor in multivariable models. Items or questionnaires addressing depressive symptoms, neuropathic-like pain, cognitive dysfunction, or catastrophizing also retained their significant association with pain factor at 3 months in both bivariate correlations and multivariable regression models adjusted for age and sex (Supplementary Table 2, available at http://links.lww.com/PR9/A158). In further exploratory analysis including programme type (PT or MDT) as a predictor variable in the multivariable model, baseline CMT remained significantly associated with follow-up pain factor (β = 2.47, *P* = 0.04), whereas programme type did not predict pain outcome (β = 0.08, *P* = 0.96).

**Table 6:** Multivariable models exploring the relationship between baseline measurements of indices of centrally facilitated pain and pain severity at 3-mo follow-up.

Baseline variable (primary predictor)	Follow-up pain factor				
	Bivariate		Adjusted for age, sex, and baseline pain factor		
	B	*P*	β	SE	*P*
QST					
PPT (kPa)	−1.00	0.15	−0.79	0.66	0.24
Adjusted *R*^2^*(P)*	—		**0.22 (<0.0001)**		
TS (0–10)	4.97	0.65	−1.87	10.10	0.85
Adjusted *R*^2^*(P)*	—		**0.21 (0.0001)**		
CPM (kPa)	−0.01	0.49	−0.01	0.01	0.60
Adjusted *R*^2^*(P)*	—		**0.21 (0.0001)**		
Widespread pain					
WPI (yes/no)	**3.79**	**0.004**	2.34	1.23	0.06
Adjusted *R*^2^*(P)*	—		**0.24 (0.0001)**		
CMT					
CMT	**4.01**	**0.0003**	**2.50**	**1.23**	**0.04**
Adjusted *R*^2^*(P)*	—		**0.25 (<0.0001)**		

B values represent bivariate regressions between baseline variables and follow-up pain factors, whereas β-values represent standardised regression coefficients for each listed baseline variable within multivariable regression models created for each central pain hypersensitivity index. Each multivariable model was adjusted for baseline pain factor, age, and sex. Multicollinearity testing yielded VIF values ranging from 1.01 to 1.68 for all independent variables indicating not significant multicollinearity between them. Values are calculated from paired baseline and follow-up data from n = 87 participants. All *P*-values have been corrected for multiple comparisons (Benjamini–Hochberg). Values in bold indicate statistical significance.

CMT, Central Mechanisms Trait; CPM, conditioned pain modulation; PPT, pain pressure detection threshold; QST, quantitative sensory testing; TS, temporal summation; WPI, Widespread Pain Index.

## 4. Discussion

We show that a Central Mechanisms Trait factor derived from 8 distinct self-reported items taken to indicate centrally facilitated pain was associated with pain severity, both at baseline and 3 months after participation in CBT-based PT or MDT programmes for people with CLBP. Observed associations remained significant after adjustment for possible confounding factors, including baseline pain severity, indicating that CMT might represent a barrier to improvement in pain during CBT-based interventions involving physiotherapy.

Multiple central mechanisms may increase chronic pain severity. Associations between discrete indices of centrally facilitated pain suggest a coordinated central nervous system response which can augment chronic pain. PPT at a site distant from tissue damage or index site of pain may reflect the integrated effect of spinal and brain nociceptive processing. TS might more directly reflect spinal sensitisation^28^ and CPM descending inhibitory control of nociceptive pathways.^6^ Supraspinal processing plays key roles^37^ and involves complex cerebral functions such as cognition, emotion, motivation, and localisation.^54^ We have shown that 8 characteristics such as anxiety, depression, catastrophizing, neuropathic-like pain, fatigue, sleep disturbance, pain distribution, and cognitive impact are associated with pain severity and each contributes to a single CMT factor in people with chronic low back pain. Central Mechanisms Trait was associated with pain severity and with PPT distant to the index site of pain, consistent with an index of centrally facilitated pain. Similar findings have been reported in chronic knee pain,^1,27^ suggesting that CMT may have generalisable validity across diagnoses.

Widespread chronic pain and sensitivity at sites beyond the area of primary pathology suggests augmented central pain processing. Widespread pain distribution predicts worse pain outcomes in fibromyalgia, a condition that is associated with centrally facilitated pain in the absence of overt peripheral tissue pathology, and in nonspecific low back pain.^64,73^ Widespread Pain Index was developed to help classify people with fibromyalgia,^74^ but WPI was not significantly correlated with QST evidence of pain hypersensitivity in our participants with CLBP. Similarly, WPI was less strongly associated with PPT distal to the knee than were other distribution indices in people with knee pain.^1,27^

We found that not all indices of centrally facilitated pain displayed significant associations with pain severity in people with CLBP. CMT was associated with pain severity both in cross-sectional and longitudinal analyses. WPI was significantly associated with pain in multivariable models only in cross-sectional analysis, and baseline QST was not significantly associated with pain severity. Different aspects of central pain facilitation might have different consequences for pain and pain prognosis in people with CLBP.

Central Mechanisms Trait is derived from items that are taken to measure diverse aspects of central pain hypersensitivity; anxiety, depression, neuropathic pain, fatigue, cognitive dysfunction, pain distribution, catastrophizing, and sleep disturbance, based on theoretical models, and by analogy with equivalent measures in preclinical models and chronic knee pain.^1,7,76^ These characteristics might be driven by overlapping mechanisms within the central nervous system.^1^ Amplified stimulus-evoked pain is associated with altered function of brain regions that monitor pain's emotional and cognitive-evaluative aspects.^51^ Negative effect is associated with worse low back pain outcomes.^8,23,26,42^ Fatigue might also drive central pain hypersensitivity and pain severity.^12,52,66^ Fatigue and sleep disturbance may be interrelated,^47^ and sleep dysfunction or deprivation can increase centrally facilitated pain by impairing central pain inhibition and prolonging motor restlessness.^41,65^ Poor sleep is a risk factor for developing LBP^10^ and has been associated with worse LBP outcomes.^4^ Brain regions involved in cognition are closely linked with pain processing.^48,69^ Cognitive-evaluative dimensions of pain, pain modulation, and anticipation involve the prefrontal cortex.^9,38,68^ Persistent nociceptive inputs compete with other sensory inputs, compromising limited neurophysiological resources, impairing cognition, and driving centrally facilitated pain.^17,29^ Neuropathic pain results from neuronal pathology,^75^ and its qualities may reflect central mechanisms.^15,21^ Neuropathic characteristics in people with LBP might indicate nerve root irritation or centrally facilitated pain and predict worse pain outcomes.^63^ Our finding that each of these diverse characteristics loads onto a single CMT factor, suggests that they indicate a shared or coordinated neurophysiological phenomenon, in addition to any unique effects of each characteristic on pain processing. This CMT factor might measure a continuous trait that underlies nociplastic^39^ rather than nociceptive or neuropathic pain classification.

Our study is subject to several limitations. Direct measurement of neuronal activity within the central nervous system is not possible within a clinical setting. We instead used several indices of centrally facilitated pain, some of them devised in populations suffering from conditions other than LBP. Findings might be different in other populations or therapeutic contexts, and exploratory modelling is warranted in larger populations to identify additional traits or mechanisms that might influence CLBP. We derived a pain factor by combining self-reported measures of pain severity to capture the complex and subjective nature of pain.^22,30,72^ However, each item displayed different loadings on pain factor. Different aspects of pain might be differently affected by central pain facilitation. Lost to follow-up was low (10.3%) in this study but still limits power in regression modelling. Unmeasured variables might confound or explain our observed associations. Despite statistical adjustments to reduce type II errors, our analyses should be viewed as exploratory, requiring confirmation in a larger independent sample.

In conclusion, we provide evidence that central mechanisms beyond those captured by QST could longitudinally influence self-reported pain severity of individuals with CLBP. Central pain facilitation might be a barrier to pain improvement within the context of CBT-based group interventions that include physiotherapy. Future research might explore possible causal relationships underlying our observed associations, whether central facilitation might differentially affect different interventions and whether a barrier to treatment response might be lifted by additional pharmaceutical or nonpharmaceutical interventions that can reduce central pain facilitation.

## Disclosures

The authors have no conflicts of interest to declare.

## Appendix A. Supplemental digital content

Supplemental digital content associated with this article can be found online at http://links.lww.com/PR9/A158.

## Supplementary Material

SUPPLEMENTARY MATERIAL
