# Supplementary material for: An observational study of centrally facilitated pain in individuals with chronic low back pain
Source: Pain Rep. 2022 Apr 13;7(3):e1003. doi: 10.1097/PR9.0000000000001003 (PMC9012603; doi:10.1097/PR9.0000000000001003)
Supplement: SUPPLEMENTARY MATERIAL [file painreports-7-e1003-s001.pdf]

Supplementary Methods

2.1. Participants and Study design

The questionnaire booklet included self-reported tools about pain severity, pain distribution, neuropathic pain indicators, negative affect, fatigue, catastrophizing and disability. All participants were invited to complete an identical questionnaire booklet at follow up, either at completion of the programme, or, for those who did not attend the follow-up appointment, by mail. All participants were invited to follow-up irrespective of the number of intervention sessions that they attended.

2.2. Therapeutic context

Patients with intermediate characteristics were allocated to one or other programme based on discussion between members of the multidisciplinary team and patient. For PT, participants were invited to attend the Back Pain Unit or community setting for approximately 4 hours on one day of each of 5 consecutive weeks (total contact time = 20 hours). For MDT, participants were invited to attend for 7 hours on one day of each of 10 consecutive weeks (total contact time up to 70 hours). The MDT included workshop sessions delivered by a multidisciplinary team of physiotherapists, clinical psychologists, occupational therapists and nurses. PT and MDT interventions were delivered in groups of up to 12 participants per programme, and aimed to address chronic pain mechanisms, anatomy, goal-setting techniques, graded exercise and pacing, stress management, challenging negative thoughts, relaxation, imagery and mindfulness as well as communication skills and medication use [2]. Programmes were delivered in an interactive, face:face, seminar format where discussions and activities were combined with group exercise, and personalised meetings with a clinician. Participants were actively encouraged to voice their questions and share their past experiences, using real-life examples. Participants were prompted through open ended questions to solve day to day problems associated with their condition. All participants were allowed to continue their usual care or pursue other management strategies throughout their programme.

2.5.1. Quantitative Sensory Testing

2.5.1.1. Forearm Pressure Pain Detection Threshold (PPT)

The testing site was the brachioradialis muscle, approximately 5 cm distal to the lateral epicondyle [6]. The handheld algometer was featured on an electronic data collection unit connected to a laptop where the amount of applied pressure was displayed on the screen. Each participant was

asked to press a button on a device held in their dominant hand as soon as the sensation of pressure became painful, thereby electronically storing the pressure value (kPa) on the computer, and simultaneously triggering an audible signal at which the examiner stopped applying pressure. The procedure was initially applied for familiarisation purposes on the dominant forearm (training site), then repeated a few minutes later on the forearm of the non-dominant arm (testing site) [6].

2.5.1.2. Temporal Summation (TS)

The tip of the blunt needle was disinfected between individuals with 2% Chlorhexidine in 70% Alcohol. For familiarisation purposes, punctate stimulation was initially applied on the non-dominant forearm. For testing, participants were asked to close their eyes and maintain their relaxed position. The 10cm VAS was anchored at left by `no pain/sharpness,’ and right by `worst imaginable pain/sharpness’. During rating of the 10 repeated stimuli, participants were asked to rate the experienced intensity of pain or sharpness with sight of their original rating after the single stimulus. A few minutes gap separated the two TS tests. Testing process was conducted after the participant reported that their skin at the test site felt normal to them.

2.5.1.3. Conditioned Pain Modulation (CPM)

The 11-point NRS had 0 anchored as “no pain” and 10 “the worst pain imaginable”.

2.5.2. Pain distribution

The 24-sites of the topographically coded manikin were right or left chest, shoulder, arm, elbow, forearm, hand, thigh, knee, leg, or foot, and head, neck, abdomen, and spinal axis. The Widespread Pain Index (WPI) classification criteria are based on pain shading over at least 4 of 5 regions (left or right upper limb, left or right lower limb, or axis (neck, upper or lower back)).

2.5.3. Central Mechanisms Trait (CMT)

The eight items have each been found to contribute to a single CMT factor in people with knee pain, with good internal consistency and association with PPT evidence of pain hypersensitivity distal to the affected joint [1]. This suggests a link between such items and centrally facilitated pain. To classify participants according to their pain distribution, we considered that one quarter

of individuals are anticipated to demonstrate evidence of centrally facilitated pain in CLBP [7], as in other populations with chronic musculoskeletal pain [4; 5; 8].

2.6. Clinical characteristics

In the painDETECT questionnaire, participant responses regarding the course, radiation and quality of their pain contributed to a total score (min. 0, max. 38), with higher scores indicating higher likelihood of neuropathic pain.

In the Hospital Anxiety and Depression Scale (HADS), anxiety and depression subscales each have possible ranges from 0 to 21, with higher scores indicating greater anxiety or depression. was assessed with the Pain Catastrophization Scale (PCS), catastrophization is measured via answering 13 questions with possible answers ranging from 'not at all' (0 points) to 'all the time' (4 points) and possible total scores from 0 to 52. Higher scores indicate higher levels of catastrophizing.

In the Fatigue Severity Scale (FSS), participants were asked to indicate their agreement with 9 statements, each on an 8-point scale (1-strong disagreement, 7-strong agreement), giving a possible summated score from 7 to 63, with higher values indicating higher levels of fatigue.

In the Roland-Morris Disability Questionnaire (RMDQ), participant agreement with 24 statements regarding their ability to perform certain activities (dressing, housework, walking) or functions (sleep) contributed to a total score (min. 0, max. 24). Higher scores indicate greater disability.

In the Fibromyalgia Severity Scale (FMSS), participant responses regarding pain location on body manikin, symptom severity at 3 questions about tiredness, sleep and forgetfulness on a 4-point scale (0-no problem, 3-severe) and whether they experienced headaches, depression or abdominal pain amongst 37 other symptoms were used to calculate a total score (min: 0, max: 31), with higher scores indicating greater severity of fibromyalgia-like symptoms.

2.7. Analysis

Distributions of data and of residuals in regression models were evaluated by Shapiro-Wilk normality testing. Where necessary, data were logarithmically transformed before analysis after or without addition of smallest measured value where appropriate. Differences were assessed with paired or unpaired Wilcoxon signed-rank tests, or independent 2-group Mann-Whitney U Tests. The Effect Size was calculated as the difference between baseline and follow-up measurements divided by baseline SD [3].

Supplementary Figures

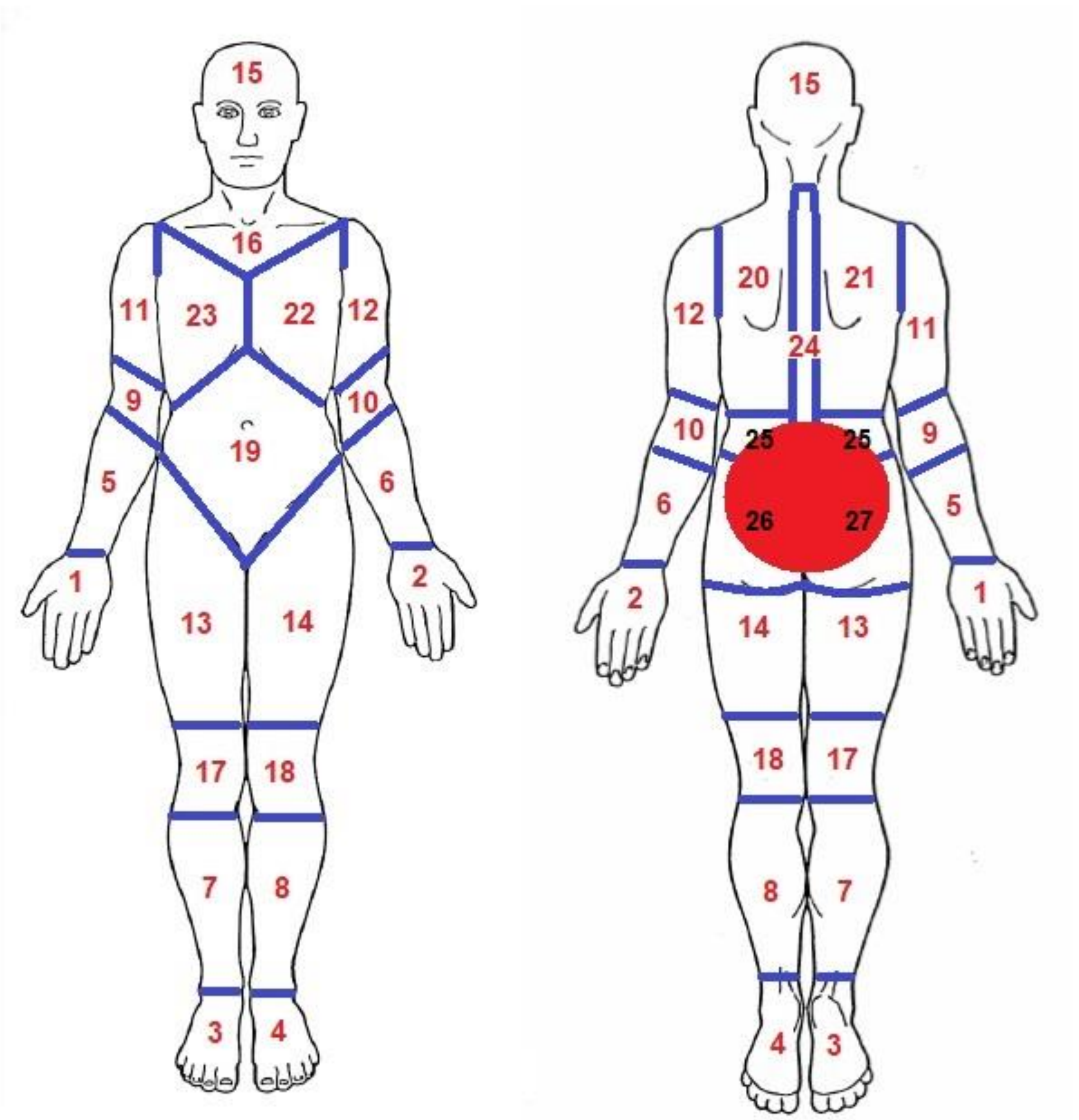

Supplementary Figure 1. Depiction of discrete diagrammatic manikin scoring based on 24 anatomical sites.

Classifications are made based on the number of painful sites the pain is distributed other than the main area of pain (lower back and lumbosacral region).

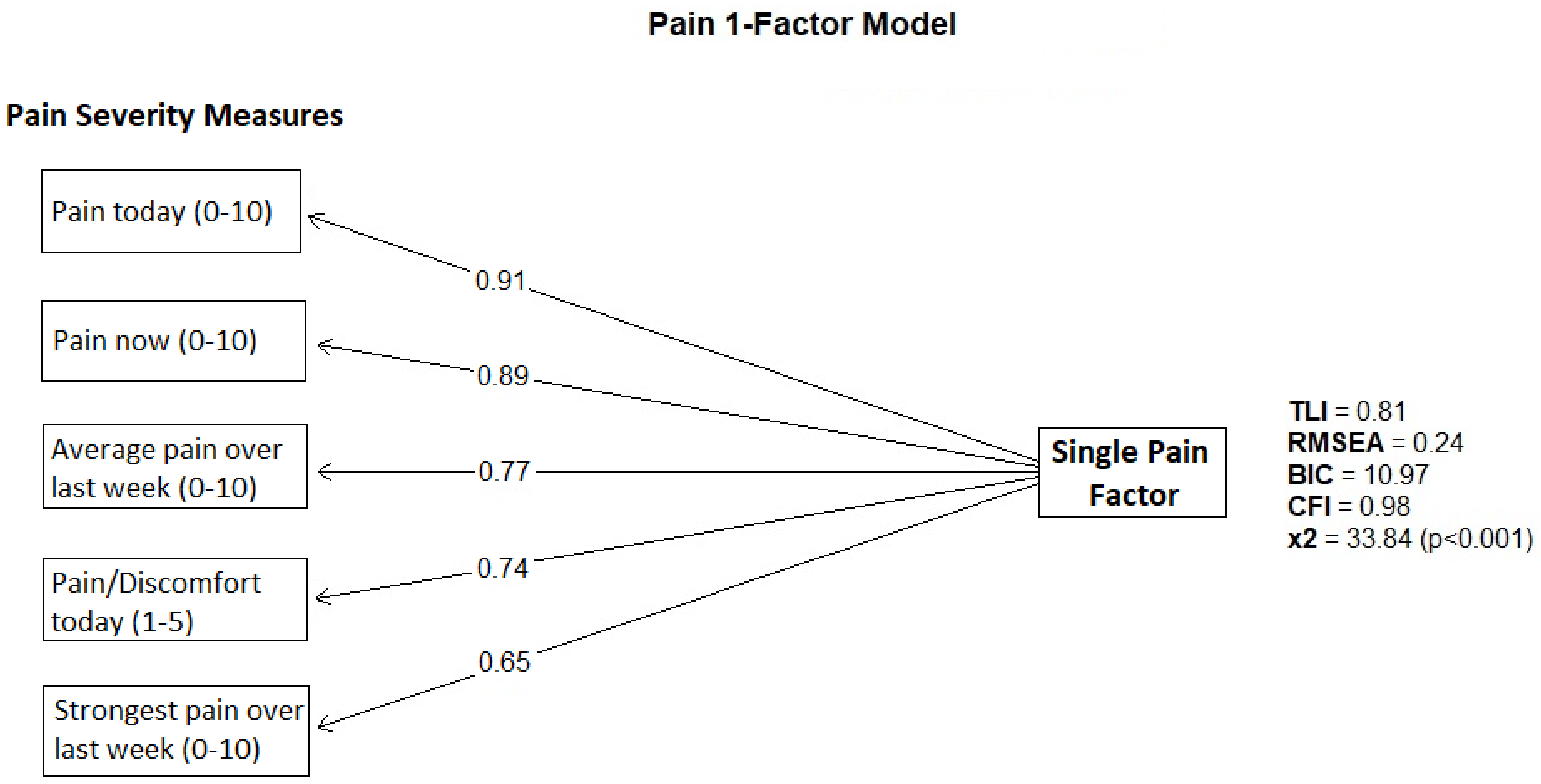

Supplementary Figure 2. Confirmatory Factor Analysis model demonstrating the factor loadings for each distinct pain severity component onto a single Pain Factor as well as statistics demonstrating the overall model fit.

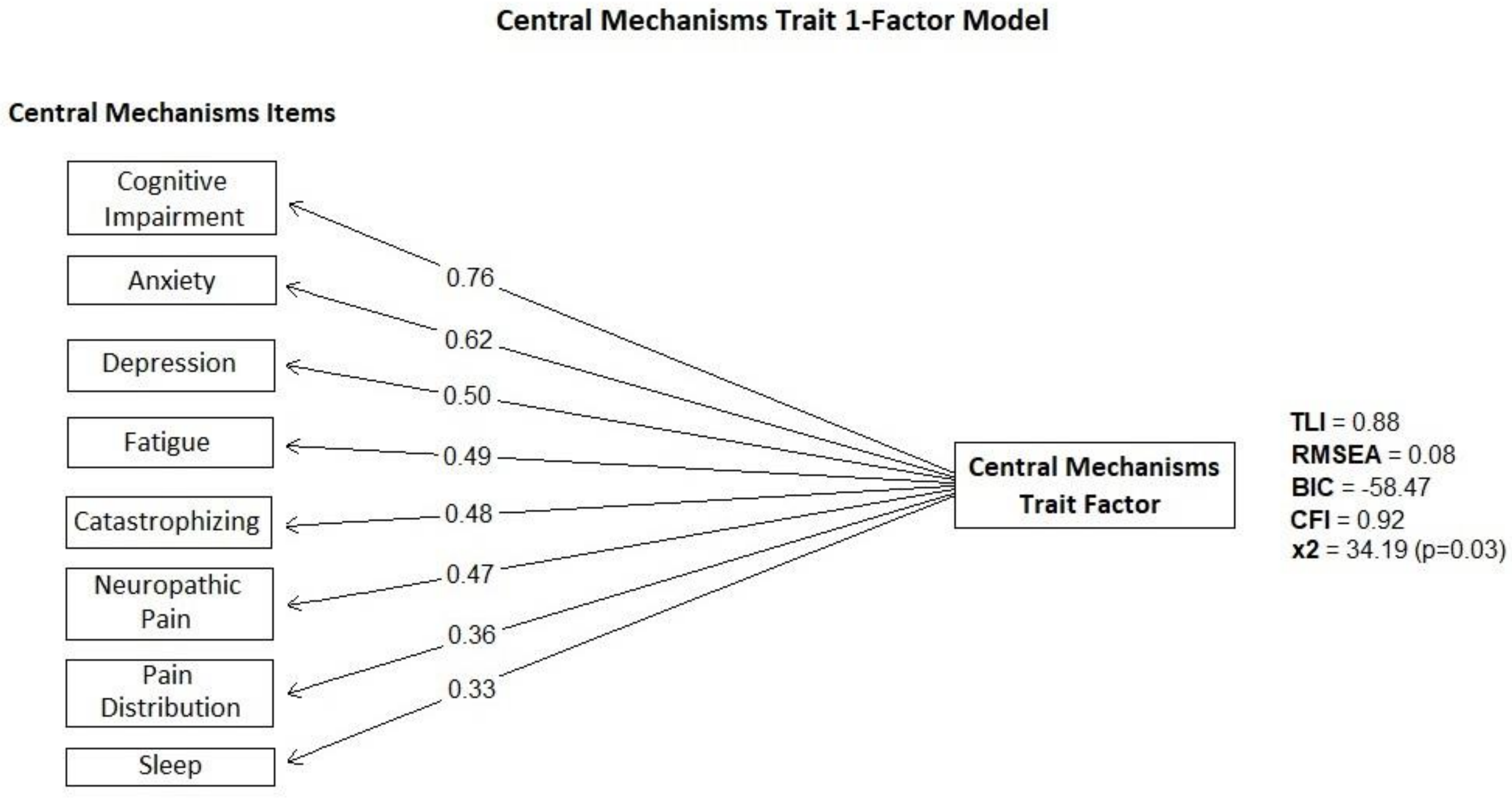

Supplementary Figure 3. Confirmatory Factor Analysis model demonstrating the factor loadings for each distinct central mechanisms components onto a single Central Mechanisms Trait Factor as well as statistics demonstrating the overall model fit.

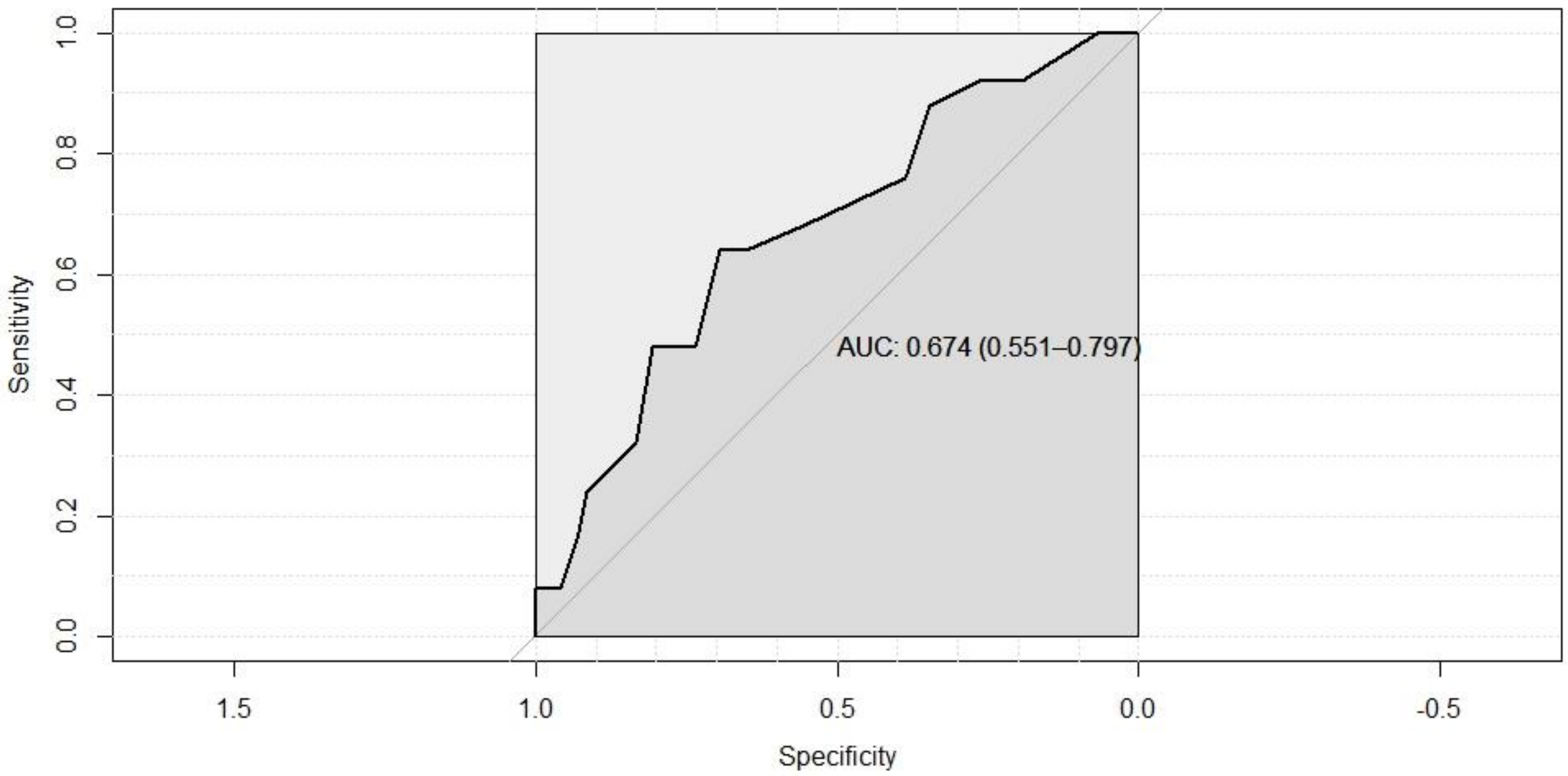

Supplementary Figure 4. Area Under the Curve (AUC) graph showing that the 24-site quantification approach adequately predicts low PPT (gain-of-function) in the forearm.

Supplementary Tables

Supplementary Table 1. Correlation matrix for demographic and anthropometric variables with indices of centrally facilitated pain and pain at baseline.

Central indices and anthropometric variables at baseline			Age (y)		Female Sex		BMI (kg/m^2)	
			Cor	p-value	Cor	p-value	Cor	p-value
Baseline	Central Indices	**PPT** (kPa)	-0.08	0.44	**-0.32**	**<0.01**	-0.02	0.77
		**TS** (0 to 10)	0.11	0.29	0.11	0.29	0.07	0.51
		**CPM** (kPa)	-0.07	0.50	-0.15	0.15	-0.01	0.92
		**WPI** (yes/no)	-0.09	0.38	0.16	0.12	0.01	0.91
		**CMT** (Index)	**-0.37**	**<0.01**	**0.21**	**0.04**	0.15	0.15
	**Pain**	**Pain Factor**	-0.08	0.43	-0.05	0.61	0.10	0.34

BMI: Body Mass Index, **CMT:** Central Mechanisms Trait, **Cor:** Pearson or Spearman Correlation, **CPM**: Conditioned Pain Modulation, **EQ4** $_{\textbf{Pain/Discomfort}}$: EQ5D5L Pain/Discomfort Domain, **kPa:** kiloPascals, **NRS:** Numerical Rating Scale, **PD** $_{\textbf{Average}}$: painDETECT Average Pain Scale (past 4-weeks), **PD** $_{\textbf{Now}}$: painDETECT Pain Now Scale, **PD** $_{\textbf{Strongest}}$: painDETECT Strongest Pain Scale (past 4-weeks), **TS:** Temporal Summation, **WPI:** Widespread Pain Index

All p-values have been corrected for multiple comparisons (Benjamini-Hochberg).
Values in **bold** indicate statistical significance (p<0.05)

Supplementary Table 2. Multivariable models exploring the relationship between baseline indices of centrally facilitated pain and the pain severity factor at baseline and at 3-months follow-up.

	**Multivariate Model**	**Baseline Pain Factor** (n=97)					**Follow-up Pain Factor** (n=87)				
		**Bivariate**		**Adjusted for age and sex**			**Bivariate**		**Adjusted for age and sex**		
		**Cor**	**p**	**β**	**SE**	**p**	**Cor**	**p**	**β**	**SE**	**p**
**Central Mechanisms Characteristic**	Anxiety (0-3) †	**0.22**	**0.03**	**1.22**	**0.55**	**0.03**	0.15	0.18	1.20	0.70	0.09
	Adjusted R^2(p)	-			0.03 (0.12)		-			0.01 (0.33)	
	Depression (0-3) †	**0.39**	**<0.0001**	**2.05**	**0.59**	**0.001**	**0.29**	**0.01**	**2.24**	**0.76**	**0.004**
	Adjusted R^2(p)	-			**0.10 (0.01)**		-			**0.07 (0.03)**	
	Neuropathic-like Pain (0-5) †	**0.39**	**<0.0001**	**1.44**	**0.35**	**<0.0001**	**0.49**	**<0.0001**	**2.10**	**0.41**	**<0.0001**
	Adjusted R^2(p)	-			**0.14 (0.001)**		-			**0.22 (<0.0001)**	
	Fatigue (7-63) †	**0.34**	**0.001**	**0.14**	**0.04**	**0.001**	0.17	0.11	0.08	0.05	0.12
	Adjusted R^2(p)	-			**0.11 (0.004)**		-			0.0004 (0.39)	
	Cognitive Dysfunction (0-3) †	**0.38**	**0.0002**	**2.05**	**0.52**	**0.0002**	**0.29**	**0.01**	**1.99**	**0.68**	**0.01**
	Adjusted R^2(p)	-			**0.12 (0.001)**		-			**0.07 (0.03)**	
	Pain Distribution (yes/no) †	0.18	0.06	**2.54**	**1.14**	**0.03**	0.11	0.31	1.15	1.45	0.43
	Adjusted R^2(p)	-			0.03 (0.12)		-			0.01 (0.75)	
	Catastrophizing (0-4) †	**0.37**	**0.0002**	**1.62**	**0.39**	**<0.0001**	**0.34**	**0.001**	**1.66**	**0.50**	**0.001**
	Adjusted R^2(p)	-			**0.14 (0.001)**		-			**0.09 (0.01)**	
	Sleep (yes/no) †	**0.24**	**0.02**	**3.14**	**1.35**	**0.02**	0.04	0.80	0.74	1.74	0.67
	Adjusted R^2(p)	-			0.04 (0.10)		-			0.01 (0.86)	

Cor values represent baseline correlations between baseline variables and baseline pain factor, whereas β-values represent standardised regression coefficients for each listed baseline variable within multivariable regression models created for each characteristic contributing to Central Mechanisms Trait. Each multivariable model was adjusted for age and sex. Multicollinearity testing yielded VIF values ranging from 1.02 to 1.19 for all independent variables indicating not significant multicollinearity between them. Values calculated from baseline data of n=97 participants. All p-values have been corrected for multiple comparisons (Benjamini-Hochberg).

† Primary predictor.
Values in **bold** indicate statistical significance.

Supplementary Table 3. Relationship between baseline indices of centrally facilitated pain and follow up pain severity or change from baseline to follow-up.

Baseline variable (Primary Predictor)		Change in Pain (Baseline to Follow-up) Adjusted for age, sex and baseline pain factor		
		**β**	**SE**	**p**
**QST**	PPT (kPa)	-0.79	0.66	0.24
	Adjusted R^2(p)		**0.14 (0.02)**	
	TS (0-10)	-1.87	10.10	0.85
	Adjusted R^2(p)		**0.12 (0.01)**	
	CPM (kPa)	-0.01	0.01	0.60
	Adjusted R^2(p)		**0.13 (0.01)**	
**Widespread Pain**	WPI (yes/no)	2.34	1.23	0.06
	Adjusted R^2(p)		**0.16 (0.001)**	
**CMT**	CMT	**2.50**	**1.23**	**0.04**
	Adjusted R^2(p)		**0.16 (0.001)**	

CMT: Central Mechanisms Trait, **CPM**: Conditioned Pain Modulation, **PPT:** Pain Pressure detection Threshold, **QST:** Quantitative Sensory Testing, **TS:** Temporal Summation, **WPI:** Widespread Pain Index

β-values represent standardised regression coefficients for each listed baseline variable within multivariable regression models created for each central pain hypersensitivity index. Each multivariable model was adjusted for baseline Pain Factor, age and sex. Multicollinearity testing yielded VIF values ranging from 1.01 to 1.68 for all independent variables indicating not significant multicollinearity between them. Values calculated from paired baseline and follow up data from n=87 participants. All p-values have been corrected for multiple comparisons (Benjamini-Hochberg).
Values in **bold** indicate statistical significance.

Supplementary Methods References

[1] Akin-Akinyosoye K, Frowd N, Marshall L, Stocks J, Fernandes GS, Valdes A, McWilliams DF, Zhang W, Doherty M, Ferguson E, Walsh DA. Traits associated with central pain augmentation in the Knee Pain In the Community (KPIC) cohort. Pain 2018;159(6):1035.

[2] Cassidy EL, Atherton RJ, Robertson N, Walsh DA, Gillett R. Mindfulness, functioning and catastrophizing after multidisciplinary pain management for chronic low back pain. Pain 2012;153(3):644-650.

[3] Copay AG, Subach BR, Glassman SD, Polly Jr DW, Schuler TC. Understanding the minimum clinically important difference: a review of concepts and methods. The Spine Journal 2007;7(5):541-546.

[4] Lluch E, Torres R, Nijs J, Van Oosterwijck J. Central sensitization in patients with osteoarthritis pain: A systematic literature review. Pain Practice 2014;14:34-35.

[5] Mlekusch S, Schliessbach J, Camara RJA, Arendt-Nielsen L, Juni P, Curatolo M. Do central hypersensitivity and altered pain modulation predict the course of chronic low back and neck pain? The Clinical journal of pain 2013;29(8):673-680.

[6] Rolke R, Baron R, Maier Ca, Tölle T, Treede R-D, Beyer A, Binder A, Birbaumer N, Birklein F, Bötefür I. Quantitative sensory testing in the German Research Network on Neuropathic Pain (DFNS): standardized protocol and reference values. Pain 2006;123(3):231-243.

[7] Smart KM, Blake C, Staines A, Thacker M, Doody C. Mechanisms-based classifications of musculoskeletal pain: part 1 of 3: symptoms and signs of central sensitisation in patients with low back (±leg) pain. Manual therapy 2012;17(4):336-344.

[8] Sterling M, Jull G, Vicenzino B, Kenardy J. Sensory hypersensitivity occurs soon after whiplash injury and is associated with poor recovery. Pain 2003;104(3):509-517.
